# Prevention of trigeminocardiac reflex-induced severe bradycardia during cerebral aneurysm clipping surgery by topical anesthesia of the dura surface and atropine administration: a case report

**DOI:** 10.1186/s40981-021-00493-1

**Published:** 2022-01-07

**Authors:** Akari Yoshida, Takafumi Seki, Yuichi Aratani, Tadashi Tanioku, Tomoyuki Kawamata

**Affiliations:** Department of Anesthesiology, Wakayama Medical University, 811-1 Kimiidera, Wakayama, 641-0012 Japan

**Keywords:** Trigeminocardiac reflex, Severe bradycardia, Dura mater

## Abstract

**Background:**

Trigeminocardiac reflex (TCR) by stimulation of the sensory branch of the trigeminal nerve induces transient bradycardia and hypotension. We report a case in which light mechanical stimulation to the dura mater during brain surgery induced severe bradycardia.

**Case presentation:**

A 77-year-old woman with bradycardia-tachycardia syndrome was scheduled for clipping of an unruptured left middle cerebral artery aneurysm. General anesthesia was performed with propofol, remifentanil, and rocuronium. Before starting surgery, the function of the pyramidal tract was examined by motor evoked potential. Transcranial electric stimulation for motor evoked potential induced atrial fibrillation and tachycardia. Continuous administration of landiolol was started and verapamil was used for tachycardia. During detachment of the dura mater from the bone, an electrocardiogram suddenly showed sinus arrest for 6 s. Immediately after the manipulation was interrupted, a junctional rhythm appeared. However, light touch to the dura mater induced severe bradycardia again, and atropine was therefore administered. In addition, the dura surface was anesthetized with topical lidocaine infiltration. After that, light touch-induced bradycardia was prevented.

**Conclusions:**

We experienced a case of severe bradycardia during surgery due to TCR caused by light mechanical stimulation to the dura mater. Topical anesthesia of the dura surface and atropine administration were effective for preventing TCR-induced bradycardia.

## Background

Trigeminocardiac reflex (TCR) is a brainstem reflex caused by stimulation of the sensory branch of the trigeminal nerve. TCR increases parasympathetic activity, resulting in bradycardia, hypotension, apnea, and increased intestinal motility [1]. TCR is known as a cause of bradycardia and hypotension during craniofacial and dental surgeries [2, 3]. We report a case in which light mechanical stimulation to the dura mater during surgery induced severe bradycardia.

## Case presentation

**Fig. 1:**
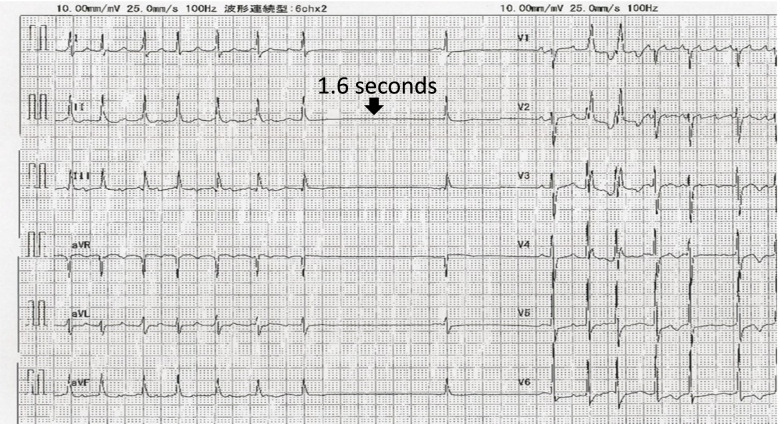
Preoperative ECG during the attack. The ECG showed bradycardia-tachycardia syndrome with irregular RR intervals, atrial flutter waveform, and sinus arrest lasting about 1.6 s

Written informed consent was obtained from the patient for this case report. A 77-year-old woman (148 cm, 59 kg) was admitted to our hospital to undergo cerebral aneurysm clipping surgery for an unruptured left middle cerebral artery aneurysm. She had bradycardia-tachycardia syndrome and received a bisoprolol patch for palpitation due to tachycardia. She had no history of syncopal attack due to bradycardia. Preoperative laboratory tests showed no electrolyte abnormalities. The preoperative electrocardiogram (ECG) during the attack showed irregular RR intervals, atrial flutter waveform, and sinus arrest lasting about 1.6 s (Fig. 1).

In the operating room, an ECG, noninvasive blood pressure, invasive radial artery pressure, percutaneous oxygen saturation, and rectal temperature were monitored. Motor evoked potential (MEP) by transcranial electrical stimulation was planned during surgery. The ECG before induction of anesthesia showed a normal sinus rhythm with a heart rate (HR) of 75 beats/min. General anesthesia was induced with propofol and remifentanil and then the trachea was intubated following administration of rocuronium. Anesthesia was maintained with propofol (2.2 μg/ml via target-controlled infusion), remifentanil (0.3 μg/kg/min), and oxygen-in-air gas mixture. Tachycardia due to tracheal intubation or head pin insertion did not occur.

**Fig. 2:**
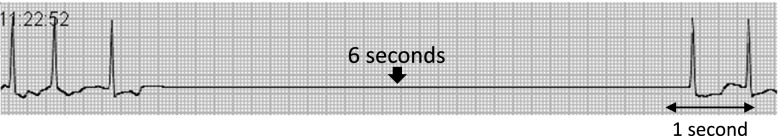
ECG during detachment of the dura mater from the bone. During detachment of the dura mater from the bone, ECG suddenly showed sinus arrest for 6 s. Immediately after the manipulation was stopped, junctional rhythm appeared

Before surgery, transcranial electrical stimulation for MEP was performed at a pulse duration of 0.5 ms, an interstimulus interval of 2 ms, and maximum stimulation intensity up to 150 mA using a train of five monophasic constant current pulses with bilateral C3/C4 (above the parietal bone). Transcranial electric stimulation induced atrial fibrillation with HR of around 170 beats/min followed by a decrease in blood pressure to 70/40 mmHg. Administration of phenylephrine increased blood pressure, but the tachyarrhythmia persisted. Continuous administration of landiolol was started and HR decreased to about 150 beats/min. Since systolic blood pressure was maintained at about 150 mmHg, surgery was started. Verapamil at 5 mg in addition to landiolol was administered for tachycardia, and HR decreased to around 100 beats/min. Approximately 30 min after verapamil administration, during detachment of the dura mater from the bone, ECG suddenly showed sinus arrest for 6 s (Fig. 2). Immediately after the manipulation was stopped, junctional rhythm appeared, followed by atrial fibrillation with HR of 100 beats/min. During the interruption of manipulation of the dura mater, gentle placement of a cotton pad on the dura mater for hemostasis induced cessation of atrial fibrillation and severe bradycardia (HR of 15 beats/min) due to junctional rhythm. Continuous administration of landiolol (7 μg/kg/min) was discontinued, the dose of remifentanil was reduced, and transcutaneous pacing pads were set. The surgery was resumed since HR returned to about 70 beats/min. However, light touch to the dura mater induced severe bradycardia (HR of 35 beats/min) again. Atropine 0.5 mg was administered and the dura surface was anesthetized with topical lidocaine infiltration (3 ml of 1% lidocaine). Lidocaine was infiltrated on the dura mater at the pterional craniotomy site from several burr holes using a syringe of 5 ml with an outer cylinder of an 18-gauge indwelling needle.

The manipulation was resumed after HR had returned to about 75 beats/min. After atropine administration and topical anesthesia of the dura surface, severe bradycardia due to dura manipulation was not observed throughout the surgery. Transcutaneous pacing was not used. After that, surgery was uneventfully completed.

Postoperatively, the patient was transferred to the general ward in stable condition without any sign of arrhythmia. The use of a bisoprolol patch was resumed on postoperative day (POD) 1. On POD 2, atrial fibrillation occurred, and HR increased to about 150 beats/min, but there were no chest symptoms. Anticoagulation therapy was started on POD 5, and the patient was discharged on POD 13.

## Discussion

In our case, light mechanical stimuli to the dura mater induced severe bradycardia. Since topical application of lidocaine to the dura surface prevented bradycardia, it was speculated that it was the response via TCR. This reflex pathway activates parasympathetic vagal neurons of the sinus node or atrioventricular node of the heart, resulting in bradycardia [4]. When TCR was defined as a decrease of HR lower than 60 beats/min accompanied by hypotension with a decrease in mean blood pressure of 20% or more, the incidences of TCR and cardiac arrest due to TCR were 11% and 2.4%, respectively, in cases of surgery in the cerebellopontine angle [3]. It has also been reported that cardiac arrest due to TCR occurs in 2.3% of cases of unruptured cerebral aneurysm clipping surgery [5]. Therefore, TCR-induced bradycardia is a symptom that should be dealt with as soon as possible. Predisposing factors for TCR include hypoxemia, hypercapnia, preoperative use of beta-blockers or calcium channel blockers, light anesthesia, children, and narcotic use [6]. Additionally, remifentanil has been reported to decrease the threshold for vagal excitation [7]. In our case, preoperative use of beta-blockers, intraoperative use of calcium channel blockers (verapamil), and intraoperative use of remifentanil were considered as predisposing factors.

When encountering severe bradycardia due to TCR, the first step of treatment is interruption of the manipulation. If the bradycardia or hypotension persists, atropine should be administered. If atropine is not effective, adrenaline administration may be considered [8]. In addition, nerve blocks and topical anesthesia of the dura mater may be effective. In ophthalmic surgery, peribulbar or retrobulbar block has been reported to reduce the incidence and severity of TCR [8]. In brain surgery, Chigurupati et al. reported a case showing the effectiveness of a trigeminal nerve block by a local anesthetic at the site where it branches from the brainstem for preventing TCR-induced bradycardia [9]. However, the use of local anesthetics near the brainstem can lead to irreversible complications [10]. It has also been reported that topical anesthesia of the dura mater with lidocaine-soaked gelfoam or lidocaine infiltration was effective for preventing TCR [11]. The dura mater was anesthetized by infiltrating 1–2 ml of 1% lidocaine with adrenaline between the leaves of the dura adjacent to the middle meningeal artery with a 25-gauge needle and tuberculin syringe in addition to covering with small gelfoam soaked in lidocaine 1%.

In our case, topical anesthesia of the dura surface with lidocaine prevented bradycardia induced by light mechanical stimuli to the dura mater. In previous reports, tetracaine, cocaine, and bupivacaine were used as local anesthetics [11]. We thought that lidocaine was more suitable because of its rapid effect. These methods may be a good way to suppress TCR without affecting the brainstem.

The limitation of this case report is that it was not determined which is more useful for preventing TCR-induced bradycardia since topical anesthesia of the dura surface and atropine administration were performed simultaneously. In addition, since there have been many reports showing that atropine and local anesthesia are effective in preventing bradycardia due to TCR [2, 4, 8, 10, 11], the novelty of our case may be limited. Regarding local anesthesia, local anesthetics were used for nerve block in most reports, and there has been only one report on topical anesthesia of the dural surface for preventing TCR-induced bradycardia. Therefore, we think that our case has educational value to make many anesthesiologists aware of the usefulness of topical anesthesia of the dural surface for preventing TCR-induced bradycardia.

## Conclusions

We experienced a case of severe bradycardia during surgery due to TCR caused by light mechanical stimulation to the dura mater. Topical anesthesia of the dura surface and atropine administration were effective for preventing TCR-induced bradycardia.

## Data Availability

Not applicable

## Abbreviations

TCR: Trigeminocardiac reflex
ECG: Electrocardiogram
MEP: Motor evoked potential
HR: Heart rate
POD: Postoperative day
